# Extra-Pleural Pneumonectomy (EPP) in Children and Adults with Locally Advanced Sarcoma: A CanSaRCC Study

**DOI:** 10.3390/curroncol29060340

**Published:** 2022-06-15

**Authors:** Caroline Rodrigues, Hagit Peretz Soroka, Agostino Pierro, Reto M. Baertschiger, Marcelo Cypel, Laura Donahoe, Derek S. Tsang, John Cho, Marc De Perrot, Thomas K. Waddell, Abha A. Gupta

**Affiliations:** 1 Division of Medical Oncology, Canadian Sarcoma Research and Clinical Collaboration (CanSaRCC), Princess Margaret Cancer Centre—University Health Network, Toronto, ON M5G 2C1, Canada; carolinemary.rodrigues@uhn.ca (C.R.); hagit.peretz@uhn.ca (H.P.S.); 2 Division of General Surgery, Hospital for Sick Children, University of Toronto, Toronto, ON M5G 1X8, Canada; agostino.pierro@sickkids.ca (A.P.); reto.baertschiger@sickkids.ca (R.M.B.); 3 Division of Thoracic Surgery, Toronto General Hospital—University Health Network, University of Toronto, Toronto, ON M5G 2C1, Canada; marcelo.cypel@uhn.ca (M.C.); laura.donahoe@uhn.ca (L.D.); marc.deperrot@uhn.ca (M.D.P.); tom.waddell@uhn.ca (T.K.W.); 4 Radiation Medicine Program, Princess Margaret Cancer Centre, University of Toronto, Toronto, ON M5G 2C1, Canada; derek.tsang@rmp.uhn.on.ca (D.S.T.); john.cho@rmp.uhn.on.ca (J.C.); 5 Division of Hematology/Oncology, Hospital for Sick Children, University of Toronto, Toronto, ON M5G 1X8, Canada

**Keywords:** sarcoma, extra-pleural pneumonectomy, thoracic surgery, radiation

## Abstract

**Simple Summary:**

Extra-pleural pneumonectomy (EPP) involves the removal of the parietal and visceral pleura, ipsilateral lungs, pericardium, and hemi diaphragm. In patients with advanced sarcoma in the pleura, EPP is often the only option for local control. The aim of our study was to review our institutional experience with EPP. Of ten patients in our study, five were alive without disease at last follow-up after multi-modality therapy including EPP. Two patients had local recurrence and died of progressive disease. One patient died of brain metastasis, one patient died of radiation induced sarcoma, and one patient died of surgical complications. Our results suggest that EPP is a feasible option for patients when used in combination with chemotherapy and radiation. Moreover, high-volume cancer centers should discuss the use of EPP during tumor board discussion.

**Abstract:**

Sarcoma can present as locally advanced disease involving pleura for which extra-pleural pneumonectomy (EPP) may be the only surgical option to ensure adequate local control. Data were collected on patients who underwent EPP between January 2009 and August 2021 at Princess Margret Hospital and SickKids (Toronto) using the CanSaRCC (Canadian Sarcoma Research and Clinical Collaboration). Ten patients with locally advanced sarcoma involving the pleura, aged 4 to 59 years (median 19.5 years) underwent EPP. Nine (90%) received pre-operative chemotherapy and eight (80%) achieved an R0 resection. Hemithoracic radiation was administered preoperatively (*n* = 6, 60%) or postoperatively (*n* = 4, 40%). Five (50%) patients were alive without disease at last follow-up (median 34.2 months) and time from EPP to last FU was median 29.2 months (range 2.2–87.5). Two patients (20%) had local recurrence, 4.3 and 5.8 months from EPP, and both died from progressive disease, 13.1 and 8.2 months from EPP, respectively. One patient died from brain metastasis (17 months), one died from radiation associated osteosarcoma (66 months), and one died from surgical complications (heart failure from constrictive pericarditis). EPP offers a feasible and life-prolonging surgical consideration for patients with locally advanced sarcoma involving the pleura in combination with chemotherapy and radiation. Consequently, EPP should be considered during multi-disciplinary tumor board discussions at high-volume centers.

## 1. Introduction

Sarcoma is a rare cancer of the soft tissue or bone, and while over 70 subtypes have been identified, sarcomas comprise approximately 2% of adult cancers [1]. Among children, soft tissue sarcoma represents approximately 7% of all childhood malignancies [2]. Sarcoma can present as a locally advanced disease involving pleura where extra-pleural pneumonectomy (EPP) is often the surgical option of choice to maximize local control for both pediatric and adult patients [3,4]. The EPP procedure involves removal of the parietal and visceral pleura, ipsilateral lung, pericardium, and hemidiaphragm [3]. EPP was first described in 1949 as a procedure used in patients with pulmonary tuberculosis [5]. Over the years, it has also been used for mesothelioma, lung cancer, thymoma, primary sarcoma, and pleuro-pulmonary metastasis from extra thoracic malignancies [3,5,6,7].

Currently, the EPP procedure is most often a part of a multimodal therapeutic approach [3] in conjunction with chemotherapy and/or radiation therapy (RT) and can offer patients long-term control [7]. The primary objective of this study is to report our EPP experience in the treatment of pediatric and adult patients with locally advanced sarcoma as well as add to the existing body of literature around EPP as part of the multimodal treatment plan in individuals with advanced sarcoma involving the pleura.

## 2. Materials and Methods

### 2.1. Data Collection, Ethics and Analysis

Data were retrospectively collected on ten consented patients who underwent EPP between 1 January 2009 and 1 August 2021, at University Health Network (Toronto General Hospital/Princess Margret Hospital and the Hospital for Sick Children in Toronto). Data were collected with approval of Institutional Review Boards. Patient demographics, treatment, and outcome details, including surgical details and complications, were extracted from the CanSaRCC (Canadian Sarcoma Research and Clinical Collaboration) prospective database. Recurrence was defined as relapse or metastatic progression of the patient. If disease was defined as recurrent distant disease, patients were characterized as having sarcoma in the pleura, distant from their primary tumor site. The median and interquartile range was used to summarize continuous variables. Frequencies and proportions were used for categorical characteristics.

### 2.2. Study Population

All patients were diagnosed with advanced sarcoma involving the pleura defined as direct pleural invasion. Patients were considered for EPP if there was lack of extra-thoracic disease, lack of contralateral disease, and adequate performance status to be able to tolerate an EPP. All consecutive patients who underwent EPP for sarcoma are included in this review. For all patients, the EPP procedure was part of a multimodal therapeutic approach in conjunction with chemotherapy and/or radiation therapy (RT). The indication for EPP as local therapy was discussed in a multidisciplinary tumor board attended by surgery, radiation, and medical oncology, radiology, and pathology.

Patients were treated with multi-modality therapy including chemotherapy that was histology specific and always doxorubicin-based. Hemithoracic radiotherapy (RT) planning for patients was done using an intensity modulated RT (IMRT) or volumetric arc therapy (VMAT) technique, using Pinnacle (Philips, Amsterdam, The Netherlands) or RayStation (RaySearch Laboratories, Stockholm, Sweden). All patients were treated using photon RT. EPP was performed by experienced surgical oncologists (AP, RB, MC, MD, TW, LD).

## 3. Results

### 3.1. Demographics

Ten sarcoma patients diagnosed with locally advanced disease involving the pleura and treated with EPP, between the ages of 4 and 59 years (median 19.5 years), were identified (Table 1). Diagnoses for each patient is listed in Table 1. Five (50%) patients had traditionally chemo-sensitive histologies including rhabdomyosarcoma (RMS), Ewing sarcoma (EWS), and pleuropulmonary blastoma (PPB). The other patients had undifferentiated pleomorphic sarcoma (UPS), synovial sarcoma (SS), adenosarcoma, and low-grade fibromyxoid sarcoma. Six patients were receiving EPP for primary disease and four for a metastatic relapse distant from their primary disease.

### 3.2. Surgical Details

EPP was performed by experienced surgical oncologists (AP, RB, MC, MD, TW, LD). Patient 9 (Table 1) developed bleeding that required ICU admission. This patient’s bleeding stopped after blood transfusions alone without the need for further surgery. The patient’s recovery was further complicated by constrictive pericarditis from the pericardial patch causing fatal heart failure 7 months after EPP. Patient 10 had post-operative complications that involved a post-pneumonectomy space empyema that necessitated out of her wound.

Eight patients had negative (R0) margins, and two patients had positive margins (R1/2) (Table 1). Patient 1 had R1 positive margins in the anterior, posterior, and lateral diaphragm as well as gross disease in the pericardium. The second patient with positive margins, Patient 4, had microscopic positive margins in the pericardium only.

Nodal status was reported in five of ten patients and was negative. In the other five, nodal status was not mentioned in surgical pathology reports or imaging.

### 3.3. Adjunctive Therapies

For all cases, EPP was used as part of a multimodal therapeutic response in conjunction with chemotherapy and/or radiation therapy (RT). Nine patients received pre-operative chemotherapy. The patient with a low-grade fibromyxoid sarcoma did not receive chemotherapy because of low grade histology. Eight out of nine (89%) had a partial response whereas one patient (Patient 9) had a mixed response (the patient had some areas of tumor that responded, and others that grew). Chemotherapy regimens included: vincristine/doxorubicin/cyclophosphamide alternating with ifosfamide/etoposide (*n* = 2), doxorubicin/ifosfamide (*n* = 4), vincristine/doxorubicin/ifosfamide/etoposide (*n* = 1), vincristine/doxorubicin/ifosfamide/actinomycin (*n* = 1), ifosfamide/etoposide (*n* = 1) (Table 2).

**Figure 1:**
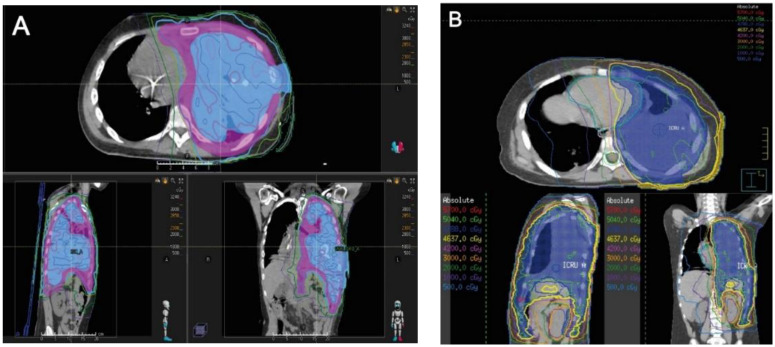
High-resolution images of the radiotherapy plans of one representative patient that received preoperative RT and one representative patient that received postoperative RT. (**A**) High-resolution images of Patient 4 who received pre-operative hemithorax RT for synovial sarcoma. A simultaneous integrated boost plan was created; 25 Gy in 5 fractions was prescribed to the entire pleural space (in magenta colorwash), while a dose of 30 Gy in 5 fractions was prescribed to the gross tumor (in blue colorwash). The thin colored lines represent regions receiving at least the dose noted in the legend; for example, the thick magenta line represents regions receiving at least 28.5 Gy, while the thick light blue line represents regions receiving at least 23 Gy. Please note the RT plan minimizes dose to the contralateral right lung. (**B**) High-resolution images of Patient 5 who received post-operative hemithorax RT for Ewing sarcoma. The planned dose was 50.4 Gy in 28 fractions to the entire pleural space (in blue colorwash). The thin colored lines represent regions receiving at least the dose noted in the legend. Similar attempts were made to minimize dose to the contralateral lung.

In addition, all ten patients received RT, six of which occurred within 2 weeks of EPP. Among these six patients, five received pre-operative RT as a short course high dose hypofractionated regimen (Figure 1) and one received post-operative RT (Table 2, Figure 1). Of the remaining four patients, one received postoperative RT approximately 2.3 months after EPP, one received pre-operative RT approximately 1 month prior to EPP, and two patients received postoperative RT approximately 1.5 months after EPP.

### 3.4. Outcome

At median time to last FU from EPP of 15 months (range 2.2 to 87.5), two patients (UPS/R0, SS/R1) had local pleural recurrence 4.3 and 5.8 months from EPP, respectively. Both died from progressive disease 13.1 and 8.2 months from EPP, respectively. The remaining eight patients did not have local recurrence at last FU. Among these eight patients, five (50%) patients were alive without disease at last follow-up (median 34.2 months) and time from EPP to last FU was median 29.2 months (range 2.2–87.5). One patient died from brain metastasis 17 months from EPP, one died from RT-associated sarcoma 66 months from EPP, and one died from heart failure secondary to a post-surgical complication of constrictive pericarditis 7 months from EPP.

## 4. Discussion

EPP is a rare procedure primarily used to treat patients with mesothelioma [3,8]. In 1976, Butchart and colleagues first used the EPP surgical approach on patients with malignant pleural mesothelioma (MPM) [6]. Conclusions of this paper suggested that EPP could be considered an appropriate approach for cases with stage I pure epithelial type [6]. In 2011, Treasure and colleagues studied EPP as a treatment option for patients with mesothelioma. Their MARS (Mesothelioma and Radical Surgery) trial reported high mortality rates of EPP (compared to no EPP), suggesting EPP did not benefit MPM patients [8]. However, more recently, our group at Princess Margaret reported better experience with a new approach of Surgery for Mesothelioma After Radiation Therapy (SMART), which consists in the delivery of a short course of hemithoracic radiation with 25–30 Gy followed by EPP the following week. This approach was performed with a 30-day mortality of 1% and achieved a disease-free survival of nearly 4 years in epithelioid node negative mesothelioma [9]. Based on this positive experience, we have been exploring this approach in other contexts, such as recurrent thymoma and our sarcoma experience reported here. With respect to the role of EPP for pediatric and adult patients with sarcoma, the literature is limited, dominated by cases of synovial sarcoma [4,10,11,12,13].

In this smaller series, we noted no 30- or 90-day mortalities. One patient developed a significant empyema and one patient developed fatal pericarditis. However, eight of ten patients underwent surgery with a straightforward post-operative course. Due to the retrospective nature of this review, we were not able to use a prospective standardized scale of reporting peri and postoperative complications. We conclude that surgery is feasible and safe and further exploration of its safety and efficacy is warranted. EPP for sarcoma should only be considered in high-volume centers with excellent results and experience with EPP for other indications.

In this study, both pediatric and adult patients were included. Our results are consistent with the Flores et al., 2006 study and with the Hameury et al., 2021 study, suggesting that EPP is a feasible procedure when used with curative intent in pediatric patients [4,14].

There were a variety of histologies included in this series, all of whom received neoadjuvant or adjuvant chemotherapy (except patient with LGFMS) and RT before and/or after EPP. Certainly, EPP alone would be considered insufficient without multi-modality therapy. Patients with RMS, EWS, and PPB are all considered relatively ‘chemo-sensitive’ sarcoma subtypes. The only patients who experienced local recurrence had an undifferentiated pleomorphic sarcoma and synovial sarcoma. These recurrences occurred relatively quickly (<6 months) post-EPP in both patients [1,7]. Whether this combined approach should be considered for these less chemo-sensitive sarcoma subtypes is uncertain. In this series, the patients with chemo-sensitive subtypes fared better than those with less chemo-sensitive subtypes, however, the total number of patients is small.

All patients were seen at a quarternary care center with surgical expertise in EPP as well as in sarcoma management. All patients were discussed multiple times at multi-disciplinary tumor boards that included members from surgery, radiation, medical oncology, pathology, radiology, and nursing. Treatment plans including number of cycles of chemotherapy, timing of RT, and surgery were discussed with careful review of radiology weighing the pros and cons of each step.

The ideal timing of RT remains unknown and patients in this series were treated with both preoperative and postoperative RT. Preoperative RT is preferred when surgical resection is certain, since it minimizes radiotoxicity to abdominal organs rising into the field postoperatively. It has theoretical advantages to target or boost the dose to areas of tumor bulk although both patients with local recurrence had preoperative radiation. We continue to explore its role.

The majority of these patients had pleural involvement as part of their primary disease, but we have also used it on highly selected patients with metastatic disease. Note the very long disease-free interval in these patients prior to consideration of this tri-modality approach. This series is too small to confirm the role of this aggressive tri-modality approach for metastatic/recurrent sarcoma, but we do note that none of these patients has succumbed to new metastases during follow up and our longest surviving patient (F/U 87 months) had recurrent disease as the indication for EPP.

## 5. Conclusions

In conclusion, EPP in combination with chemotherapy and either pre- or postoperative RT is feasible and appears to be safe in patients with primary or metastatic/recurrent sarcoma. This series is too small to confirm its true efficacy but given that it may be the only alternative when treating patients with advanced sarcoma involving the pleura, we propose to continue this approach and expand to additional patients. These complex and rare cases benefit from multidisciplinary tumor board discussion to ensure proper patient selection and sequencing with other modalities (like chemotherapy and RT). Hopefully, this study will encourage referrals to high-volume centers for patients who have locally advanced sarcoma. Future directives can consider how EPP influences respiratory function post-surgery. Moreover, studies could investigate the nature of chemo-sensitive subtypes of sarcoma compared to other sarcoma subtypes. Future studies will benefit from a longer follow-up period to better assess the long-term implications off EPP in all patients.

## Figures and Tables

**Table 1:** Diagnosis and outcome of patients who underwent EPP.

Patient	Age at Diagnosis of Pleural Disease (Years)	Diagnosis	Type of Disease, DF1 (m)	Time from EPP to Last FU (m)	Status at Last FU	Resection Margins
1	22	RMS	Primary	17.0	Deceased (brain mets)	R1 Multiple resection margins positive (diaphragm, pericardium).
2	59	UPS	Recurrent distant disease (DFI = 27.7 m)	13.1	Deceased: (recurrent pleural disease)	R0
3	18	Adenosarcoma	Recurrent distant disease (DFI = 40.6 m)	87.5	ANED	R0
4	19	SS	Primary	8.2	Deceased (recurrent pleural disease)	R1: Focal positive margin at pericardium
5	13	EWS	Primary	66	Deceased (RAS)	R0
6	4	RMS	Recurrent distant disease (DFI = 40.3 m)	29.2	ANED	R0
7	4	PPB	Primary	31.2	ANED	R0
8	29	EWS	Recurrent distant disease (DFI = 188.6 m)	2.2	ANED	R0
9	36	SS	Primary	7	Deceased (heart failure)	R0
10	20	Low grade fibromyxoid sarcoma	Primary	6.4	ANED	R0

DFI = Disease free interval (time between initial diagnosis of cancer and relapse); m = months; RMS = rhabdomyosarcoma; UPS = undifferentiated pleomorphic sarcoma; SS = synovial sarcoma; EWS = Ewing sarcoma; PPB = pleuropulmonary blastoma; RAS = radiation-associated sarcoma.

**Table 2:** Chemotherapy and radiation (RT) of patients.

Patient	Pre-Op Chemotherapy	RT [Dose (Gy), Fraction]	Time to EPP from Diagnosis of Pleural Disease (Months)	Time from RT to EPP (Months)
1	VDC/IE	Post Op (60, 25)	5.5	2.3
2	Dox/Ifos	Pre-Op (39, 3)	8.8	0.4
3	Dox/Ifos	Pre-Op (30, 5)	6.9	0.3
4	Dox/Ifos	Pre-Op (30, 5)	9.7	0.3
5	VDC/IE	Post Op (50.4, 28)	4.7	0.4
6	VIDE	Pre-Op (45, Unk)	5.0	1.0
7	IVAD ×4, IVA ×2	Post Op (25, 45)	7.0	1.5
8	IE	Pre-Op (30, 5)	10.4	0.4
9	Dox/Ifos	Pre-Op (30, 5)	5.6	0.3
10		Post Op (50, 25)	1.6	1.5

## Data Availability

All data can be found in the text.
